# Cardiac cells and mesenchymal stem cells derived extracellular vesicles: a potential therapeutic strategy for myocardial infarction

**DOI:** 10.3389/fcvm.2024.1493290

**Published:** 2024-12-18

**Authors:** Dan Qin, Xiaobo Wang, Jun Pu, Houxiang Hu

**Affiliations:** ^1^ Department of Cardiology, Affiliated Hospital of North Sichuan Medical College, Nanchong, China; ^2^ Academician Workstation, Affiliated Hospital of North Sichuan Medical College, Nanchong, China

**Keywords:** myocardial infarction, cardiac cells, cell-cell communication, mesenchymal stem cells, extracellular vesicles

## Abstract

Despite improvements in clinical outcomes of acute myocardial infarction (AMI), mortality rates remain high, indicating the need for further understanding of the pathogenesis and developing more effective cardiac protection strategies. Extracellular vesicles (EVs) carry proteins and noncoding RNAs (ncRNAs) derived from different cardiac cell populations, mainly including cardiomyocytes, endothelial cells, endothelial progenitor cells, cardiac progenitor cells, cardiosphere-derived cells, immune cells, fibroblasts and cardiac telocytes have vital roles under both physiological and pathological process such as myocardial infarction (MI). The content of EVs can also indicate the status of their parental cells and serve as a biomarker for monitoring the risk of cardiac injury. Examining these vesicles can offer fresh perspectives on the development of MI and assist in creating innovative treatments. Additionally, mesenchymal stem cells (MSCs) (MSC-EVs) derived EVs have been shown to have significant potential in cardiac regeneration. In this review, we will discuss the current understanding of the role of EVs in cardiac communication, with a focus on the perspectives of EVs from various cardiac cells and MSCs for their potential uses as cardiac therapies after MI.

## Graphical Abstract

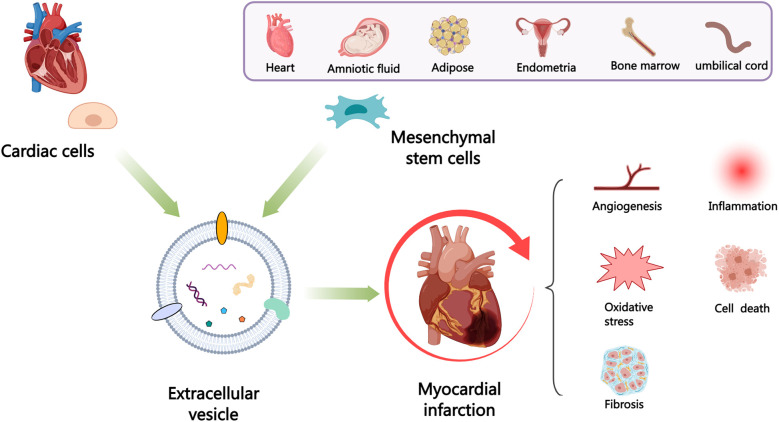

## 1 Introduction

Cardiovascular disease (CVD) continues to be a top cause of mortality globally, with acute myocardial infarction (AMI) representing one of its most critical forms (1). AMI is caused by sudden interruption of myocardial blood supply, leading to hypoxia and death of myocardial tissue, ultimately resulting in left ventricular remodeling and heart failure. The outlook for patients with ST-segment elevation myocardial infarction (MI) (STEMI) is poorer than for those with non-STEMI within 28 days following an acute coronary syndrome (ACS). However, over a decade of monitoring, the long-term mortality rates for patients with STEMI and non-STEMI were high (19.6% and 22.8%, respectively) and similar (2). Timely reperfusion treatment through percutaneous coronary intervention (PCI) has been shown to enhance the clinical outcomes for individuals suffering from AMI (3). Nevertheless, it is unable to promote regeneration and functional recovery of the damaged myocardium, and there is still ample scope to further improve the mortality rates among patients with MI. Heart transplantation is the only treatment for the latest stage of heart failure. Consequently, there is an immediate need to further enhance existing strategies or develop novel approaches to promote cardiac protection and repair.

Extracellular vesicles (EVs), containing several molecules such as proteins, lipids, and nucleic acids, have the ability to act as intercellular messengers and have the disease diagnosis and therapeutic potential. The cardiovascular system consists of various cell types, mainly including cardiomyocytes (CMs), endothelial cells (ECs), endothelial progenitor cells (EPCs), cardiac progenitor cells (CPCs), cardiosphere-derived cells (CDCs), immune cells, cardiac fibroblasts (CFs) and cardiac telocytes (CTCs) (4) that communicate via paracrine (such as EVs) or cell-cell interaction and participate in numerous cardiac physiological and pathological activities, which can be either advantageous or harmful (5). In addition, mesenchymal stem cells (MSCs) have received considerable interest owing to their potential for multilineage differentiation and ease of isolation and acquisition (6, 7). Transplantation of MSCs reportedly alleviates myocardial injury and improves cardiac function post-MI. However, it is unlikely that these benefits are solely due to the direct replacement or differentiation of MSCs into cardiac tissue, given that most transplanted cells are rapidly lost from the heart (8). Moreover, cell transplantation carries the risk of inducing rejection (9), embolism (10), calcification or ossification of the infarct area (11), and arrhythmia (12). The therapeutic benefits of MSCs are mainly due to the EVs (13), and multiple studies have shown that MSC-derived EVs (MSC-EVs) can alleviate MI through various mechanisms (14, 15).

This review aims to provide insight into EVs biogenesis, composition and uptake. Furthermore, a comprehensive review of the current knowledge about the roles of EVs released by different cardiac cell types in MI and the advancements in utilizing MSC-EVs for MI therapy. Furthermore, we highlight the major challenges that must be overcome before clinical translation and the strategies for enhancing the potency of EVs as biotherapeutics. Aiming to provide effective information for developing treatment strategies based on EVs to improve endogenous repair.

## 2 Biogenesis, composition and uptake of EVs

### 2.1 Biogenesis of EVs

**Figure 1:**
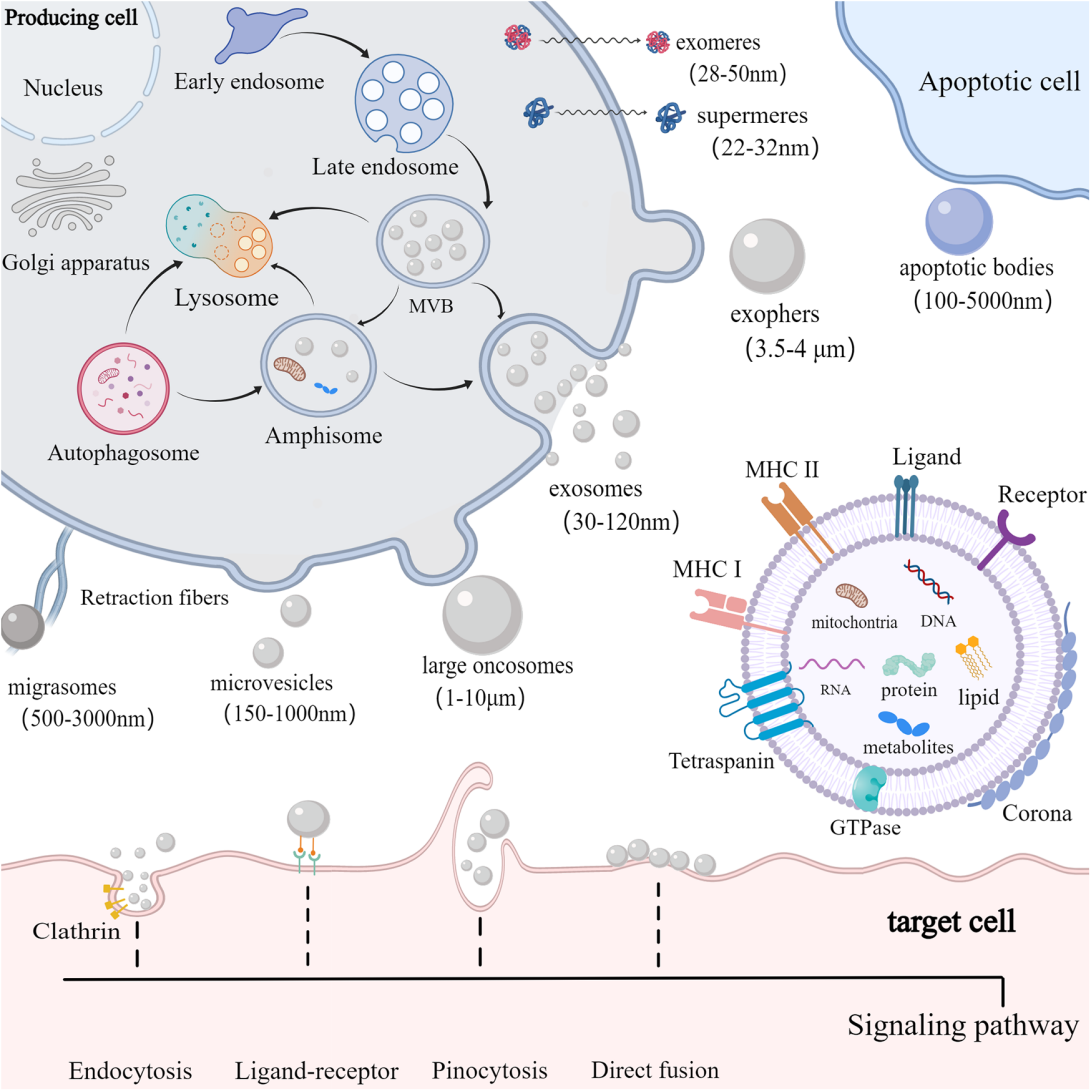
Biogenesis, composition and uptake of EVs. cells secrete EVs (including exosomes, ectosomes, and apoptotic bodies) and NVEPs (such as supermeres and exomeres) into the extracellular environment. Exosomes are produced via exocytosis, while ectosomes (such as microvesicles and large oncosomes) are formed through budding. Apoptotic bodies are vesicles formed during apoptosis. EVs contain proteins, DNA, RNA, lipids, metabolites, and mitochondria. EVs can also form a corona on their surface. The membrane of EVs predominantly includes GTPase, MHC class I and II molecules, tetraspanins like CD9, CD63, and CD81, as well as various receptors and ligands. EVs are primarily internalized by target cells via receptor-ligand interactions, clathrin-mediated and clathrin-independent endocytosis, pinocytosis, and direct membrane fusion.

EVs are small vesicles released into the extracellular space and serve as essential messengers for intercellular communication. EVs comprise proteins, nucleic acids, and lipids, and their lipid bilayer membranes protect their contents from enzymatic degradation (16). EVs are primarily classified according to their origin and biogenesis. Currently, researchers have recognized at least three primary categories of EVs: exosomes, ectosomes, and apoptotic bodies (17) (Figure 1). Exosomes, ranging from 30 to 120 nm, are formed through the inward budding of endosomal compartments (18). Ectosomes form through the outward budding of the cell membrane and primarily consist of microvesicles (150–1,000 nm), large oncosomes (1–10 μm), small ectosomes (30–150 nm), and ARRDC1-mediated microvesicles (ARMMs) (30–150 nm), which are not depicted in the illustration. Apoptotic bodies (100–5,000 nm) are generated by fragmentation of cells undergoing apoptosis. Furthermore, there are several other specialized EVs, mainly including migrasomes (500–3,000 nm) and exophers (3.5–4 μm) (19, 20). Migrasomes are emitted from the retracting fibers of cells in motion and might serve to eliminate impaired mitochondria from cells (21). Furthermore, cells are capable of releasing nonvesicular extracellular particles (NVEPs), such as exomeres (28–50 nm) and supermeres (22–32 nm) (22, 23) (Figure 1). It is believed that they transport a range of molecules including RNA, DNA, and proteins, yet the process of how supermeres and exomeres are formed remains a mystery.

Among those EVs, exosomes are mostly studied for therapy development. Exosomes are produced via a complex endocytic process, where the cell membrane invaginates to create early endosomes, which then mature into late endosomes or multivesicular bodies (MVBs) containing intraluminal vesicles (ILVs). When MVBs merge with the cell membrane, ILVs are discharged into the extracellular environment as exosomes (18). Certain MVBs may be directed to lysosomes for breakdown or merge with autophagosomes to form amphisomes. Amphisomes may be moved to lysosomes for breakdown, or directed to the plasma membrane to discharge their contents outside the cell. The intricate processes governing cargo sorting and the creation of ILVs involve both ESCRT-dependent and ESCRT-independent pathways (24). The ESCRT system, comprising four soluble protein complexes and auxiliary proteins like ALIX, VPS4, and TSG101 (25), plays a crucial role in directing proteins to ILVs and generating exosomes. In addition, the formation of MVBs can be promoted by the ESCRT-independent pathway (26). Studies have shown that the formation of MVBs could still occur in ESCRT-depleted cells (27).

EVs of different types overlap in size, and consensus has not yet emerged on specific markers of EVs subtypes, complicating their separation with existing isolation techniques like ultrafiltration, ultracentrifugation, precipitation, immunoaffinity capture, and size exclusion chromatography (28, 29). The complexity of heterogeneous mixtures of EVs and NVEPs makes their separation even more difficult. In response to the numerous types of EVs and the uncertainty of their biogenesis, the International Society for Extracellular Vesicles (ISEV) recommends adopting the general term “EV” with specific operational extensions, instead of using inconsistent and potentially confusing labels like “exosomes” and “ectosomes,” which are linked to intricate and hard-to-define biogenesis processes (30). In this review, the EVs types will not be differentiated and will be collectively called EVs. Further research is required to develop optimal techniques for segregating and characterizing distinct EVs subpopulations and improving separation of distinct EVs and NVEPs, enabling the establishment of a more accurate and specific nomenclature.

### 2.2 Composition of EVs

A single cell is capable of generating various kinds of EVs with distinct structure and biochemical properties. A conserved range of proteins are enriched in EVs, including tetraspanins (TSPANs) (CD9, CD63, and CD81), TSG101, ALIX, and some specific lipids (17). Major histocompatibility complex (MHC) molecules are enriched on EVs compared to parent cells (31). Nonetheless, the composition of EVs cargo, including proteins, nucleic acids, lipids, and organelles, as well as their membrane and corona, can differ significantly depending on their biogenesis, the source cell, cell vitality, and the culture environment (32). Microvesicles are characterized by expression of Annexin A1, Annexin A2 and *α*-Actinin 4 (17). ARMMs characteristically express ARRDC1 and TSG101 (33, 34). Large oncosomes feature enrichment of Annexin A1, ARF6, V-ATPase G1, and CK18 (16). Small ectosomes are characterized by expression of CD9 and CD147. Moreover, apoptotic EVs characteristically express Annexin V (35). Additionally, migrasomes are enriched with TSPAN4, cholesterol and integrins (36). Exophers contain protein aggregates and damaged mitochondria (20). It should be noted that CD9, CD63 and CD81 have long been used as exosome markers. However, there is growing acknowledgment that TSPAN-containing EVs can bud directly from the plasma membrane (37). According to their biogenesis, these EVs can be classified as ectosomes/microvessels (37). Interestingly, MSC-EVs also express CD29, CD44, and CD73, molecules that are surface markers of MSCs (38). Upon release into biological fluids, EVs interact with extracellular components to form a protein corona (PC) on their surface via electrostatic interactions and protein aggregation (39, 40). The route of EVs administration and the proteomic characteristics of different pathological conditions can impact the composition of the PC surrounding the EVs, which affects their physicochemical properties, biodistribution, and targeting ability (41, 42).

### 2.3 Uptake of EVs

EVs serve as a means of intercellular communication, capable of delivering diverse molecules to nearby cells or across greater distances, either through uptake or by the binding of EVs surface proteins to cell receptors. After exiting the cell, EVs are primarily internalized by target cells through interactions between receptors and ligands, clathrin-dependent and clathrin-independent endocytosis pathways, pinocytosis, and direct fusion, resulting in alterations in the physiological state of target cells (43). EVs possess features like minimal toxicity, reduced immune response, the ability to traverse biological barriers including the blood-brain barrier, and the capability to deliver cargo to target cells (44). In this context, a substantial body of evidence has recently emerged to demonstrate the therapeutic effects of MSC-EVs in a range of pathological conditions (45).

## 3 EVs derived from cardiac cells

**Figure 2:**
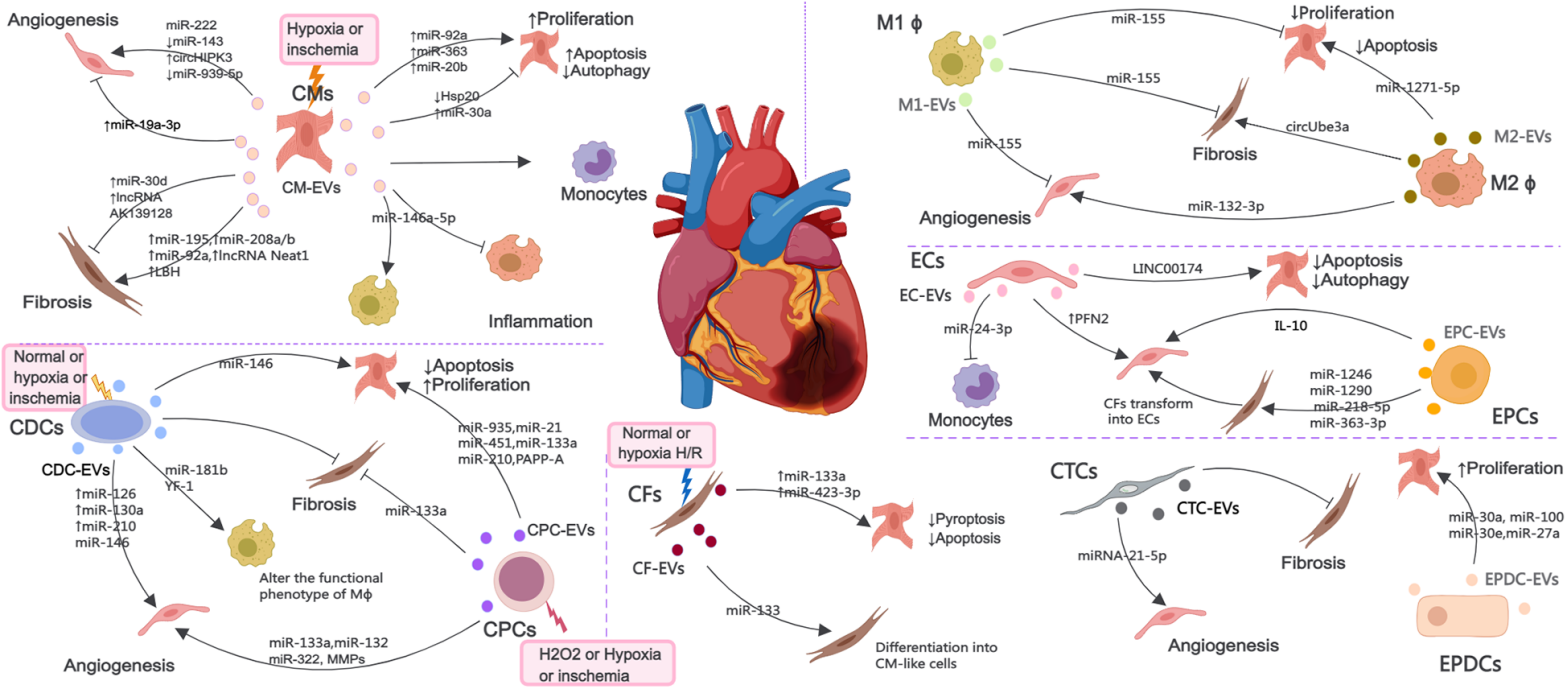
The EV-mediated cross-talk among various cardiac cells such as CMs, ECs, EPCs, CPCs, CDCs, Mϕ, CFs, CTCs and EPDCs under normal, H2O2, hypoxia or ischemia condition, which are involved in the regulation of angiogenesis, inflammatory response, cell death and myocardial fibrosis.

MI causes cardiac cell death, triggers angiogenesis and inflammatory response, induces cardiac fibrosis, and ultimately leads to myocardial remodeling and heart failure. After MI, various cardiac cells, mainly including CMs, ECs, EPCs, CPCs, CDCs, macrophages (M ϕ), CFs, CTCs and epicardium-derived cells (EPDCs), can communicate with each other through EVs to promote improvement or impairment of cardiac function (Figure 2). Considering the possible importance of EVs in the mechanisms of injury, healing and tissue remodeling post-MI, understanding EVs derived from cardiac cells holds promise for improving endogenous repair opportunities through the use of intervention strategies.

### 3.1 CM-derived EVs

EVs secreted by CMs (CM-EVs) mediate communication between cardiac cells under healthy and ischemic conditions. MicroRNAs (miRNAs) are small noncoding RNAs (ncRNAs) that control gene expression at the post-transcriptional level (46). EVs secreted by CMs cultured under hypoxic or ischemic conditions can protect cardiac microvascular ECs (CMECs) from oxidative damage and promote angiogenesis, which are attributable to miR-222, miR-143 and circHIPK3 (47–49). Interestingly, the EVs derived from CMs treated with hyperbaric oxygen can induce upregulation of long non-coding RNA MALAT1 (lncRNA MALAT) in CM-EVs to suppress miR-92a expression, thereby promoting neovascularization (50). Nonetheless, inhibition of miR-19a-3p in CM-EVs can downregulate the protein level of hypoxia-inducible factor-1α (HIF-1α) and promotes ECs proliferation and angiogenesis after MI (51). MiR-939-5p (52) levels were notably reduced in EVs from the coronary serum of myocardial ischemia patients, enhancing angiogenesis via the miR-939-inducible nitric oxide synthase (iNOS)-NO pathways, with CMs potentially being the origin of these bioactive EVs.

AMI increases the production of cardiac EVs, originating mainly from CMs and ECs. EVs accumulating in the ischemic myocardium are rapidly taken up by infiltrating monocytes and regulate local inflammation (53). MiR-146a-5p derived from CM-EVs can induce inflammation and exert anti-inflammatory effects by regulating macrophages polarization (54). EVs derived from ferroptotic CMs induce M1 macrophages (M1 ϕ) polarization and exacerbate cardiac inflammation during MI (55). The effect of CM-EVs on CFs under hypoxic or ischemic conditions seems to be inconsistent in different studies, and can promote fibrosis reversal through miR-195 (56), miR-208a/b (57), miR-92a (58), lncRNA Neat1 (59) and limb-bud and heart (LBH) (60), or inhibit fibrosis response through lncRNA AK139128 (61) and miR-30d (62).

CM-EVs can also act on CMs and regulate their survival. EVs from hypoxic CMs regulate autophagy by transferring miR-30a between CMs (63). After MI, the expression of HSP20 in CM-EVs decreases, leading to CMs apoptosis and inflammatory response (64). MiR-92a, miR-363, and miR-20b (belonging to the miR-106a-363 cluster) secreted from EVs derived from human induced pluripotent stem cell (iPSC) derived CMs (iCMs) promote CMs re-entry into the cell cycle, induce cell proliferation and improve ischemic myocardial injury (65). In addition, circulating EVs from infarcted hearts can mediate the transfer of myocardiac miRNAs to bone marrow (BM) mononuclear cells, downregulate CXCR4 expression, and increase the number of circulating progenitor cells. Therefore, infarcted hearts released EVs can induce systemic responses for cardiac repair (66).

### 3.2 EC and EPC-derived EVs

ECs and EPCs are another important source of EVs during MI and play a important role in maintaining and establishing the integrity of blood vessels. The levels of profilin 2 (PFN2) in serum and EC-EVs of patients, mice, and pigs with MI are elevated. PFN2 and EVs from PFN2-overexpressing ECs can enhance ECs proliferation, migration, and tube formation, and increase vessel numbers in infarcted myocardium (67). Khan et al. have demonstrated that EVs secreted by EPCs (EPC-EVs) can inhibit cell apoptosis, reduce scar size, and promote neovascularization after MI (68). Inflammation can impair the repair of the heart by EPC-EVs, and *interleukin-10* (*IL-10*) deficiency weakens the repair effect of EPC-EVs on infarcted myocardium by upregulating integrin-linked kinase (68). CFs have innate plasticity and can acquire CMs or endothelial phenotype upon exposure to transcription factors and other molecules (69, 70). EPC-EVs facilitate the transformation of CFs into ECs, enhance angiogenesis post-MI, and prevent myocardial fibrosis by delivering miR-1246, miR-1290, miR-218-5p, and miR-363-3p to CFs (71, 72). Recently, zhao et al. have found that coculture with EPC-EVs improved human umbilical venous ECs (HUVECs) proliferation, angiogenic and migration ability, while alleviated hypoxia-induced apoptosis *in vitro*.

Krüppel-Like Factor 2 is highly expressed in ECs under laminar flow and has anti-inflammatory effects. EVs secreted by ECs overexpressing krüppel-Like Factor 2 inhibit Ly6CHigh monocytes recruitment by shuttle miR-24-3p, improve ischemia reperfusion (I/R) injury, and alleviate cardiac inflammation (73). LncRNA 174 (LINC00174) in EC-EVs mitigate I/R-induced myocardial damage by inhibiting p53-mediated autophagy and apoptosis of CMs (74).

### 3.3 CDC and CPC-derived EVs

CDCs and CPCs have shown significant potential in promoting the regeneration and repair of damaged myocardium (75, 76). The anti-apoptosis effect of CPCs derived EVs (CPC-EVs) can be mediated by pregnancy-associated plasma protein-A (PAPP-A) (77) and various RNAs, mainly including miR-21, miR-451, miR-935, miR-133a, and miR-210 (78–82). In addition, miR-133a in CPC-EVs can improve cardiac function in a rat MI model by reducing fibrosis and hypertrophy and increasing CMs proliferation and vascularization (81). CPC-EVs can also promote ECs immigration via the degradation of extracellular matrix (ECM) (83). MiR-132 in CPC-EVs has the potential to boost angiogenesis both *in vitro* and *in vivo* by suppressing RasGAP-p120 (82). Bioengineered CPC-EVs carrying a pro-angiogenic miR-322 can increase ECs migration and capillary tube formation via increased NADPH oxidase 2 (NOX2)-derived ROS, and enhance angiogenesis in the border zones of infarcted hearts (84). EVs derived from hypoxic CPCs (H-CPC-EVs) can enhance tube formation of ECs and reduce the expression of profibrotic gene in transforming growth factor-*β* (TGF-*β*)-stimulated fibroblasts and cardiac fibrosis after I/R injury (85). The angiogenesis ability of H-CPC-EVs is highly correlated with oxygen concentration, with the angiogenesis effect being most effective at 5% O2 concentration and the angiogenesis signaling pathway at 1% O2 concentration (86). In addition, Emmert et al. have evaluated the safety, feasibility and efficacy of human derived CPC-EVs in a pig model of AMI. Intracoronary (IC) delivery of EVs reduced infarct size, improved left ventricular ejection fraction (LVEF), significantly alleviated myocardial fibrosis, and increased vascular density (87).

CDCs derived EVs (CDC-EVs) can exert cardioprotective effects by transferring miR-146 (partially beneficial), thereby reducing CMs apoptosis and promoting angiogenesis (88). EVs released by hypoxic CDCs can induce angiogenesis via enrichment of miR-126, miR-130a, and miR-210 (89). In addition, CDC-EVs also act on macrophages by transferring Y RNA fragments (YF1), enhancing the secretion of IL-10, reducing CMs apoptosis, and promoting ischemic heart repair (90). CDC-EVs can polarize M1 ϕ to a proangiogenic phenotype dependent on arginase 1 upregulation and independent of VEGF-A, which promote angiogenesis (91). CDC-EVs can modify the polarization state of macrophages by transfer of miR-181b into macrophages that inhibits proinflammatory signaling and enhances phagocytosis to promote a cardioprotective response *in vivo* (92). This helps to understand the immune regulatory mechanism of CDC-EVs in macrophages polarization after AMI. Study has revealed a mechanism for amplifying the biological activity of EVs, in which CDC-EVs promote SDF1 and VEGF secretion of fibroblasts, promote angiogenesis, and reduce scar quality after MI by promoting phenotypic transformation from inert fibroblasts to therapeutic active cells (93). In a large animal study, intramyocardial (IM) delivery of CDC-EVs was found to reduce scar formation, prevent adverse remodeling, and increase vascular density in pigs with AMI and chronic MI (CMI), but it appears to have the disadvantage of requiring IM delivery (94). In addition, CDC-EVs can inhibit ventricular arrhythmias in chronic ischemic cardiomyopathy by reducing fibrosis, eliminating slow conduction electrical pathways, and suppressing ventricular arrhythmias (95).

### 3.4 Macrophage-derived EVs

After MI, immune cells like monocytes and macrophages move to the injured site to remove dead cells. Macrophages are versatile cells within the innate immune system, essential for initiating inflammation and aiding in tissue repair following MI. In addition, it also participates in interactions with other cardiac cells to coordinate the post MI process within the heart tissue. Following MI, EVs derived from M1 ϕ (M1-EVs) deliver miR-155 to ECs, diminishing their angiogenic capacity by concurrently targeting *Rac family small GTPase* 1, *p21 (RAC1)-activated kinase 2*, *sirtuin 1* (Sirt1), and *protein kinase AMP-activated catalytic subunit alpha 2* (96), to CFs to decrease the expression of son of sevenless 1, thereby inhibiting CFs proliferation and promoting inflammation by lowering the levels of suppressor of *cytokine signaling 1* (97), and to CMs to inhibit CMs proliferation by inhibiting the IL-6R/JAK/STAT3 signaling pathway (98).

EVs derived from M2 ϕ (M2-EVs) promote angiogenesis after MI by delivering miR-132-3p to ECs and downregulating the expression of *THBS1* (99). M2-EVs can also deliver miR-1271-5p to CMs, alleviating hypoxia induced apoptosis via down-regulating SOX6 (100) and release circUbe3a into CFs, promoting proliferation, migration, and phenotype transformation of CFs by repressing RhoC, exacerbating myocardial fibrosis after AMI (101).

### 3.5 CF-derived EVs

During cardiac stress, CFs proliferate and differentiate into myofibroblasts, secreting ECM proteins and pro-inflammatory cytokines, leading to cardiac fibrosis and remodeling. CFs are both a source of cardiac protection and a carrier of disease fibrosis. EVs secreted by CFs under hypoxia/reoxygenation (H/R) can mimic the beneficial effects of ischemic post-treatment through miR-423-3p, reducing apoptosis of CMs (102) and deliver miR-133a to CMs, targeting *ELAVL1* and preventing pyroptosis caused by I/R (103). Moreover, EVs secreted by CFs (CF-EVs) can also regulate their own differentiation. MiRNA-133 in CF-EVs can promote the differentiation of CFs into CM-like cells (104). Under hypoxic conditions, multiple ECM proteins in CFs are upregulated, and CF-EVs have different effects on the viability of CMs at different stages of hypoxia and reoxygenation (105). Treatment of fibroblasts with long-term, low-dose sulforaphane can enhance the release of their anti-remodeling CM-targeted EVs, effectively reducing cardiac hypertrophy and scar size and improving cardiac function post-MI (106).

### 3.6 Other cardiac cell-derived EVs

CTCs are a type of stromal cell with elongated extensions. MiRNA-21-5p in EVs released by CTCs (CTC-EVs) can target the *cell death inducing p53 target 1* gene, which suppresses apoptosis of ECs under ischemic and hypoxic conditions, facilitating angiogenesis and regeneration following MI (107). In addition, CTC-EVs can also decrease cardiac fibrosis following MI (108). The outermost layer of the heart, known as the epicardium, can be reactivated following an injury to an adult heart. EPDCs can release EVs (EPDC-EVs) carrying miR-30a, miR-100, miR-30e, and miR-27a, promoting the proliferation of CMs after myocardial injury (109). In addition, clusterins of EVs in pericardial fluid from AMI patients improve MI by activating the epicardium, increasing arterial generation, and reducing CMs apoptosis (110).

Although some EVs are generated under normoxic conditions and cannot reflect the state of the infarcted tissue, their beneficial effects can provide us with ideas for developing new treatment strategies. The majority of research relies on EVs extracted from cells grown *in vitro*, potentially failing to represent the properties of EVs released by different cells in ischemic heart tissue *in vivo*. The seemingly opposite therapeutic effects may reflect different levels of stress on cardiac cells, and it is necessary to further elucidate the interactions between EVs from different sources in the development of MI. In addition, it is necessary to explore the components of EVs and their interactions with specific cardiac targets. This will deepen our understanding of the function of EVs and pave the way for new treatment strategies to alleviate MI and promote cardiac repair.

## 4 Biological functions of MSC-EVs in MI

### 4.1 Promotion of angiogenesis

**Figure 3:**
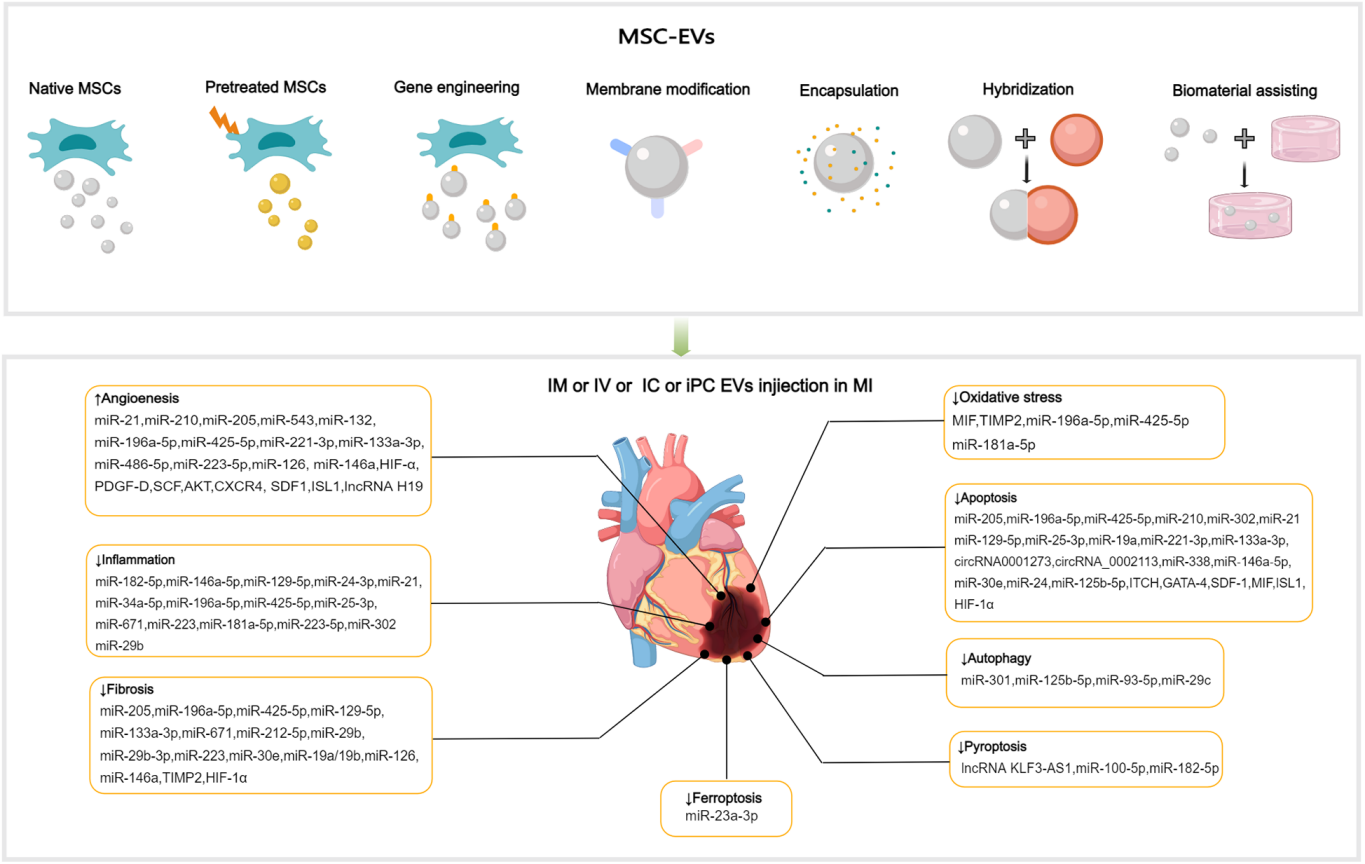
Applications of MSC-EVs in MI. MSC-EVs can be utilized in their original state and enhanced autophagy through preconditioning, gene engineering,membrane modification, encapsulation, hybridization and biomaterial-assisting. EVs can be applied via IM, IV, IC and iPC injection in MI or I/R animal models. MSC-EVs can improve angiogenesis, inflammation, cell death (such as apoptosis, autophgy, pyroptosis, ferroptosis), oxidatve stress and cardiac fibrosis after MI.

MSC-EVs contain various ncRNAs and paracrine effector molecules that promote angiogenesis (Figure 3). CMECs are derived from coronary microvessels exhibiting rapid expansion, tube formation, and proangiogenic abilities. CMECs are susceptible to damage under ischemic and hypoxic conditions. MSC-EVs containing miR-543 can enter CMECs and downregulate collagen type IV alpha 1 (COL4A1), promotes CMECs angiogenesis after MI (111). Adipose-derived MSC-EVs (ADMSC-EVs) containing miR-205 which enhance the proliferation and migration of MECs, inhibit apoptosis, reduce cardiac fibrosis, and increase angiogenesis in mice with MI (112). The role of miRNA-21 in promoting angiogenesis has been well-researched, with evidence showing that EVs from human endometrial MSCs (EnMSCs) containing miR-21 enhance microvascular density via the PTEN/AKT signaling pathway, offering better cardioprotection than those from BMMSCs or ADMSCs (113). Additionally, miRNA-132 in MSC-EVs was discovered to promote tube formation in HUVECs by suppressing the target gene *p120RasGap*, thereby boosting neovascularization in the peri-infarct region (114). Similarly, miR-210 downregulated the Efna3 gene to enhance angiogenesis and provide therapeutic benefits for MI (115). Xu et al. (116) have revealed that neonatal rat CMs (NRCMs) cultured under hypoxic conditions treated with EVs derived from BMMSCs, ADMSCs, and umbilical cord MSCs (UCMSCs) reduced apoptosis and promoted angiogenesis by increasing levels of VEGF, basic fibroblast growth factor, and hepatocyte growth factor (HGF). Notably, ADMSC-EVs exhibited the most pronounced effects. In mice with MI, IM injection of cardiac MSC-EVs was shown to promote capillary angiogenesis in the infarcted area, stimulate CMs proliferation, and improve cardiac function (117). In addition, Takov et al. have demenstrated for the first time that EVs secreted from human foetal amniotic fluid MSCs can protect hearts from I/R injury *in vivo* and markedly stimulated ECs migration *in vitro*, but did not protect isolated primary CMs in models of simulated I/R injury and were not proangiogenic *in vitro* (118). The combined effects of multiple active substances in MSC-EVs collectively regulate post-MI angiogenesis.

### 4.2 Anti-inflammation

MI can cause a strong inflammatory response, and the duration and intensity of this inflammation are closely related to the prognosis. MiR-182-5p within BMMSC-EVs can reduce inflammation and enhance heart function after MI by suppressing the TLR4/nuclear transcription factor-κB (NF-κB) signaling pathway (119) (Figure 3). High-mobility group box 1 (HMGB1) acts as a damage-associated molecular pattern (DAMP), triggering cytokine release and attracting inflammatory cells (120). The transfer of miR-129-5p through BMMSC-EVs has been shown to inhibit CMs apoptosis, cardiac fibrosis, and inflammatory response in mice with MI by targeting HMGB1 (121). Shi et al. have reported that UCMSC-EVs promote the transformation of fibroblasts into myofibroblasts within an inflammatory setting, reducing the inflammatory reaction and CMs apoptosis post-MI, while not exacerbating cardiac fibrosis (122). The transcription factor forehead box o3 (Foxo3) plays a critical role in T cells activation (123). Recent studies have revealed that intrapericardial (iPC) injection of MSC-EVs accumulate in the mediastinal lymph nodes and induce regulatory T cells (Tregs) differentiation, promoting cardiac repair. The absorption of MSC-EVs by MHC-II + APCs triggers Foxo3 activation through the PP-2A/Foxo3 signaling route. Foxo3 promotes the production of IL-10, IL-33, and IL-34, establishing a Treg-inducing niche in mediastinal lymph nodes. Ultimately, this coordination results in the resolution of inflammation and the promotion of cardiac repair post-MI (124). The observed immunomodulatory effects post-MI indicate the potential of EVs to coordinate the transition from the inflammatory to the resolution phase following ischemic injury.

Following MI, cardiac macrophages undergo a transition from proinflammatory M1 ϕ in the early stage (1–3 days) to reparative M2 ϕ, which predominate in the late stage (after 5 days) (125). This transition is crucial in limiting inflammation and facilitating cardiac repair. Through gene sequencing and bioinformatics, it was discovered that miR-24-3p within UCMSC-EVs can suppress the expression of phosphoinositide-specific phospholipase C beta 3 and activate the NF-*κ*B pathway, resulting in the promotion of M2 ϕ polarization and alleviation of inflammatory responses post-MI (126). Furthermore, ADMSC-EVs are capable of triggering the sphingosine 1-phosphate/sphingosine kinase 1/sphingosine phosphate receptor 1 signaling pathway, resulting in the polarization of M2 ϕ. This results in a reduction in local inflammation and cardiac injury following MI (127). Compared with BMMSC-EVs, EVs derived from lipopolysaccharide (LPS)-pretreated BMMSCs exhibited superior therapeutic effects in terms of promoting M2 ϕ polarization *in vitro* and alleviating post-MI inflammation and CMs apoptosis *in vivo* by mediating macrophage polarization in an MI mouse model (128). The immunomodulatory properties of ADMSC-EVs may not be constitutive but are instead induced by the inflammatory microenvironment. The immunosuppressive effect was apparent only when ADMSCs were pre-activated by proinflammatory stimuli. Pre-activated ADMSCs release EVs with higher levels of miRNAs (such as miR-34a-5p, miR-21, and miR-146a-5p) that regulate the M2 phenotype untreated EVs (129).

### 4.3 Anti-oxidative stress

Elevated oxidative stress and overproduction of reactive oxygen species (ROS) following MI intensify mitochondrial DNA damage, leading to greater myocyte injury and a subsequent rise in fibrosis and tissue remodeling (130, 131). Mitochondrial transplantation is a promising novel therapy for CVD. Mitochondrial transfer between cells can be achieved through several methods, mainly including tunneling nanotubes, EVs, and cell fusion (132). EVs facilitate the transfer of functional mitochondria to recipient cells, rescuing damaged cells through multiple pathways (133, 134). MSC-EVs were discovered to restore mitochondrial transcription factor A (TFAM) levels in recipient cells through the delivery of TFAM mRNA and mitochondrial DNA. This process prevents mtDNA damage and cytoplasmic mtDNA leakage, effectively alleviating mitochondrial damage and inflammation in acute kidney injury cells and animal models (135). MSC-EVs represent a promising avenue for the development of nanotherapies for diseases characterized by mitochondrial damage. Currently, no direct evidence demonstrates the independent functionality of mitochondria in EVs (136). It is noteworthy that damaged cells release mitochondrial DAMPs into circulation, which may have notable immune consequences. Interestingly, selective packaging of mitochondrial proteins into EVs appears to prevent this process (137, 138). The macrophage migration inhibitory factor (MIF) is essential for regulating cell homeostasis (139). Compared with BMMSC-EVs, injection of EVs derived from BMMSCs overexpressing MIF elicited superior cardioprotective effects in attenuation of CMs injury post-MI by inhibiting mitochondrial fragmentation, apoptosis, and ROS overexpression via activation of the AMPK signaling pathway (140) (Figure 3). BMMSC-EVs, which contain miR-214, can target Ca^2+^/calmodulin-dependent protein kinase II (CaMKII) to inhibit oxidative stress-related injuries in cardiac stem cells (CSCs), including apoptosis, calcium imbalance, and excessive ROS accumulation (141). In ADMSC-EVs, miR-196a-5p and miR-425-5p were found to mitigate CMs ischemia-induced mitochondrial dysfunction and excessive ROS production, increase angiogenesis, and promote M2 ϕ polarization. Furthermore, miR-196a-5p can reduce and reverse myofibroblast activation and decrease collagen production (142).

### 4.4 Cell death reduction

#### 4.4.1 Reducing cellular apoptosis and autophagy

Following MI, myocardial cells undergo apoptosis and severe autophagy, causing cardiac injury and deterioration of cardiac function. Moderate autophagy during myocardial ischemia is essential for maintaining tissue viability. The relationship between cellular autophagy and apoptosis is complex, and maintaining a balance between the two is critical for cell survival (143, 144). The process of cell apoptosis is mainly initiated by mitochondrial, death receptor, and endoplasmic reticulum (ER) pathways (145–147). BMMSC-EVs preconditioned with hypoxia reduced CMs apoptosis of rats with AMI by upregulating microRNA-24 (148) (Figure 3). MSC-EVs loaded with miR-25-3p can target pro-apoptotic genes (*FasL* and *PTEN*) and *enhancer of zeste homologue 2*, leading to decreased apoptosis in CMs and reduced inflammation in both *in vivo* and *in vitro* MI models (149). SOX6, part of the SOXD group, can amplify LPS-triggered apoptosis in CMs by stimulating the Bcl-2 family pathway (150). UCMSC-EVs can prevent CMs apoptosis and alleviate myocardial injury post-MI by transferring miR-19a to target SOX6, subsequently activating AKT and suppressing Jun N-terminal kinase 3 (*JNK3*)/caspase-3 activation (151).

Sun et al. have found that miR-221-3p derived from Aged MSC-EVs attenuated the function of angiogenesis and promoted the survival of CMs. Upregulation of miR-221-3p in aged MSCs improved their ability of angiogenesis, proliferation and migration, and reduced apoptosis via the PTEN/AKT pathway (152). Furthermore, Zhang et al. have indicated that EVs derived from young MSCs can enhance the activity of aged MSCs and improve their myocardial repair function by transferring miR-136 and downregulating apoptotic peptidase-activating factor (153). BMMSC-EVs carrying itchy E3 ubiquitin ligase can mediate ubiquitination of apoptosis signal-regulated kinase-1, leading to the inhibition of CMs apoptosis and improved myocardial injury post-AMI (154). EVs derived from UCMSCs overexpressing MIF (MIF-EVs) exert cardioprotective effects, such as the promotion of angiogenesis, inhibition of apoptosis, alleviation of cardiac fibrosis, and preservation of heart function. MIF-EVs exert their biological effects through miR-133a-3p and the subsequent activation of the AKT signaling cascade (155). MSC-EVs also contain molecules that exert destructive effects. Low miR-153-3p expression in MSC-EVs significantly boosted the activation of the angiopoietin-1/VEGF/VEGFR2/PI3 K/AKT/eNOS signaling pathway, which inhibited apoptosis in ECs and CMs while enhancing angiogenesis in an oxygen-glucose deprivation model (156). Circular RNA (circRNA) is a kind of ncRNA, involving in the development of CVD. Tian et al. demonstrated that EVs originating from circRNA_0002113-deficient BMMSCs could decrease H9C2 cell apoptosis caused by H/R and mitigate MI by by sponging miR-188-3p to regulate RUNX1 nuclear translocation. Specifically, the circRNA_0002113/miR-188-3p/RUNX1 axis mediated apoptosis by regulating the USP7/p53 pathway both *in vitro* and *in vivo* (157). CircRNA 0001273 in UCMSC-EVs can remarkably reduce myocardial cell apoptosis in ischemic environment and promote MI repair (158). Accumulation of unfolded or misfolded proteins in CMs, a condition referred to as ER stress, can cause apoptosis and fibrosis (159). Zhang et al. have found that UCMSC-EVs alleviated ER stress-induced apoptosis in H9C2 cells subjected to H/R by activating the phosphatidylinositol 3-kinase (PI3K)/AKT pathway (160). Mitogen-activated protein kinase (MAPK) is crucial for controlling cell proliferation and apoptosis. Fu et al. have found that miR-338 in MSC-EVs can inhibit CMs apoptosis in MI model rats by regulating the MAP3K2/JNK signaling pathway, thereby substantially improving cardiac function (161).

Reportedly, BMMSC-EVs carrying overexpressed miR-301 could reduce infarct area and improve cardiac function in rats with MI by inhibiting myocardial autophagy compared with BMMSC-EVs group (162) (Figure 3). The role of p53 in autophagy is contingent upon its subcellular localization in the nucleus or cytoplasm (163). Xiao et al. have demonstrated that the benefits of MSCs transplantation post-MI can be attributed to the improved autophagic flux. The mechanism of MSC-induced autophagic inhibition involves the transfer of miR-125b-5p from MSC-EVs to native cells, where it interferes with p53/B-cell lymphoma 2-interacting protein 3 signaling (164). Compared with healthy individuals, patients with AMI exhibit elevated serum levels of miR-93-5p and inflammatory factors. *In vitro* and *in vivo* experiments have shown that miR-93-5p in ADMSC-EVs can alleviate heart damage after MI by targeting autophagy-related protein 7-mediated autophagy and TLR4-mediated inflammation (165). The mammalian target of rapamycin (mTOR) is a negative regulator of autophagy. MiR-29c derived from BMMSC-EVs can target PTEN to activate the AKT/mTOR pathway, ultimately inhibiting CMs autophagy after I/R injury (166).

#### 4.4.2 Reducing pyroptosis

Pyroptosis, a proinflammatory programmed cell death process, is characterized by the disruption of cell integrity and the release of inflammatory cytokines. In a mouse model of AMI, pyroptosis was triggered within 24 h. Preventing pyroptosis has been demonstrated to significantly decrease infarct size and enhance heart performance (167). Sirt1 has been discovered to inhibit the activation of the NLRP3 inflammasome. Mao et al. have demonstrated that the lncRNA KLF3-AS1 acts as a competing endogenous RNA (ceRNA) for miR-138-5p, which regulates the expression of Sirt1. *In vitro* and *in vivo* experiments have shown that lncRNA KLF3-AS1 within MSC-EVs can modulate Sirt1, thereby preventing cell pyroptosis and reducing MI progression by functioning as a ceRNA to sponge miR-138-5p (168) (Figure 3). Proteomic analysis conducted seven days after ligating the left coronary artery revealed that treatment with MSC-EVs could substantially reduce leukocyte accumulation in the infarct area and surrounding regions and decrease the expression of low-density lipoprotein receptor-1 (LOX1), NLRP3 inflammasome, caspase-1, cleaved caspase-3, GSDMD, Bcl-2, and Bax, resulting in preservation of cardiac function (169). Liang et al. have reported that miR-100-5p in UCMSC-EVs suppresses the expression of Foxo3, inhibiting the activation of the NLRP3 inflammasome and suppressing H/R-induced CMs pyroptosis (170). Yue and colleagues have uncovered that gasdermin D (GSDMD) is robustly expressed in H/R-exposed cardiac cells and I/R-injured myocardial tissues. The upregulation of GSDMD promoted H/R-induced cardiac cell pyroptosis. Further analysis revealed that GSDMD is a miR-182-5p target. Administration of MSC-EVs carrying miR-182-5p attenuated GSDMD-dependent pyroptosis and inflammation induced by H/R, improved cardiac function, reduced MI, and decreased inflammation and pyroptosis *in vivo* (171).

#### 4.4.3 Reducing other types of cell death

Ferroptosis, an iron-dependent form of programmed cell death, is marked by the buildup of ROS, disrupted iron balance, and lipid peroxidation. *Divalent metal transporter 1* (*DMT1*), a Fe2+transporter, is known to be markedly elevated in AMI. Song et al. have showed that *DMT1* is a target gene of miR-23a-3p.Human umbilical cord blood derived MSC-EVs reduce *DMT1* levels through miR-23a-3p, thereby preventing ferroptosis and lessening heart damage, which is abolished in EVs with knocked down miR-23a-3p expression (172) (Figure 3). Recently, cuproptosis has been identified as a novel non-apoptotic cell death process triggered primarily by intracellular copper accumulation (173). A relationship between copper overload and ferroptosis has been reported (174). A recent study identified 19 differentially expressed genes related to both copper overload and ferroptosis (CFRGs) in healthy individuals and those with AMI. Further research has identified the upregulation of immune-related CFRGs (*CXCL2*, *DDIT3*, *DUSP1*, *CDKN1A*, *TLR4*, and *STAT3*) in both animal models and patients, suggesting the potential of these genes as early diagnostic biomarkers for AMI. This evidence also indicates the interplay between cuproptosis and ferroptosis pathways in the development of MI (175). Recently, wang et al. have proposed an innovative treatment strategy for MI using the circASXL1 signaling network, UCMSC-EVs effectively repairs infarcted myocardium by stimulating CMs cell-cycle reentry and cytokinesis in a circASXL1-dependent manner (176).

### 4.5 Ameliorating cardiac remodeling

MiR-671 in ADMSC-EVs can directly bind to TGF-β receptor 2 and prevent SMAD2 phosphorylation, leading to decreased cell apoptosis, inflammation, and fibrosis, thereby alleviating MI-like symptoms both *in vitro* and *in vivo* models (177) (Figure 3). Low levels of miR-212-5p expression were detected in clinical and pathological samples, as well as in animal models of MI-induced cardiac fibrosis. ADAMTS16, a disintegrin and metalloproteinase with thrombospondin motif 16, was found to activate latent TGF-β, accentuating fibrosis and cardiac function of the pressure-overloaded heart (178). BMMSC-EVs containing miR-212-5p (179) and miR-29b-3p (15) have been demonstrated to prevent myocardial fibrosis caused by MI by suppressing the NLRC5/VEGF/TGF-β1/SMAD pathway and reducing ADAMTS16 respectively. P53 is a target gene of miR-223, UCMSC-EVs containing miR-223 reduced myocardial fibrosis and inflammation in MI rat models and accelerated angiogenesis of HUVECs through the p53/S100A9 axis (180). Moreover, MSC-EVs can act directly on CFs and reduce fibrotic scar formation in the ischemic heart by regulating the secretion of fibronectin and collagen (181).

Xiao et al. have reported that BMMSC-EVs can improve heart remodeling and function after MI by modulating the balance of the RAS, specifically by upregulating ACE2-Ang1-7-Mas and downregulating the ACE-AngII-AT1R pathway, promoting the conversion of AngII to Ang1-7. This ultimately reduces Ang II-mediated adverse effects on CMs (182). The suppression of matrix metalloproteinases (MMPs) by tissue inhibitors of matrix metalloproteinase 2 (TIMP2) is essential in the remodeling process after MI. According to reports, UCMSC-EVs with high levels of TIMP2 improve heart performance by reducing oxidative stress and ECM remodeling, in part through the AKT/secreted frizzled-related protein 2 (Sfrp2) pathway (183). Compared with the use of EVs or MSCs alone, the combined delivery of EVs and MSCs (first IM injection of EVs, followed by transplantation of MSCs into the heart) further reduced the collagen area, enhanced neovascularization, reduced infarct size, and improved cardiac function. This may be attributed to EVs improving the microenvironment and facilitating the recruitment and retention of MSCs. The optimal time for continuous stem cell delivery appears to be the third day after EVs treatment (184). Likewise, the use of BMMSC-EVs as carriers to deliver exogenous miR-19a/19b to infarcted tissues combined with MSCs transplantation reduced cardiac fibrosis and substantially improved cardiac function in mice with MI (185). Recently, Tcf21 has been identified as a critical target for improving cardiac fibrosis. LncRNA-Tcf21 antisense RNA inducing demethylation (TARID) that enriched in UCMSC-EVs was identified to up-regulate Tcf21 expression. Formulated lncRNA-TARID-laden lipid nanoparticles up-regulated Tcf21 expression in EPDCs and improved cardiac function and histology after MI *in vivo* (186).

## 5 Effects improvement strategies of MSC-EVs

Despite the considerable therapeutic potential of natural MSC-EVs, limitations in their yield, targeting, on-demand delivery, and treatment feedback have hindered their widespread application (187). Therefore, it is important to improve the yield of EVs production and regulate their biological functions, current approaches including: preconditioning, gene engineering, membrane modification, encapsulation, hybridization and biomaterial-assisting (Table 1).

**Table 1:** Strategies for improving the therapeutic effect of MSC-EVs in MI.

Improvement strategy	Therapeutic agent	MSCs	Animal model	Mechanisms	Results	Reference
Preconditioning	Hypoxia	BMMSCs	Rat/AMI	Upregulated miR-24	Apoptosis↓	(148)
		MSCs	Rat/MI	Regulated PI3K/AKT and p53 signaling	Apoptosis↓, fibosis↓	(188)
	Atorvastatin	MSCs	Rat/MI	Up-regulating lncRNA H19 and its downstream pathways	Apoptosis↓, angiogenesis↑	(189)
	Hemin	MSCs	Mice/MI	Regulated HMGB1/ERK pathway	Fibrosis↓, CM senescence↓	(190)
	Tongxinluo	MSCs	Rat/AMI	Targetin IRAK1/NF-κB p65 pathway	Apoptosis↓, inflammation↓	(191)
	Tanshinone IIA	UCMSCs	Rat/MI/RI	Inhibited CCR2 activation	Angiogenesis↑, monocyte infiltration↓	(192)
	LPS	BMMSCs	Mice/MI	Suppressed NF-κB pathway and partly activated AKT1/AKT2 pathway	Inflammation↓, apoptosis↓	(128)
	CTRP9-281	CBSC	Mice/MI	Upregulated SOD2/SOD3 expression	Apoptosis↓, angiogenesis↑, fibrosis↓	(193)
	3D cell cultivation	MSCs	Rat/AMI	–	Apoptosis↓, angiogenesis↑, inflammation↓	(194)
Gene engineering	circRNA_0002113	BMMSCs	Rat/I/R	CircRNA_0002113/miR-188-3p/RUNX1 axis regulated the USP7/p53 pathway	Apoptosis↓	(157)
	miR-301	BMMSCs	Rat/MI	Overexpressed miR-301 decreased LC3-II LC3-I ratio and increased P62 expression	Autophagy↓	(162)
	cTnI-targeted short peptide	BMMSCs	Rat/MI	Inhibited the expression of *Clic5*,*Homer1* and *Hopx* genes	CMs proliferation↑	(195)
	miR-30e	BMMSCs	Rat/MI	Inhibited LOX1 expression, downregulating the activity of the NF-κB p65/Caspase-9 signaling	Apoptosis↓, fibrosis↓	(196)
	miR-486-5p	MSCs	NHP/MI	Regulated fibroblastic MMP19-VEGFA cleavage signaling	Angiogenesis↑	(197)
	TIMP2	UCMSCs	Rat/MI	Upregulated the AKT Sfrp2 pathway	Apoptosis↓, angiogenesis↑ oxidative stress↓, ECM remodeling↓	(183)
	AKT	UCMSCs	Rat/AMI	Activated PDGF-D	Angiogenesis↑	(198)
	CXCR4	BMMSCs	Rat/MI	Upregulated AKT Signaling	Angiogenesis↑, cardiac Remodeling↓	(199)
	SDF1	MSCs	Mice/MI	Activated the PI3K pathway	Autophagy↓, angiogenesis↑, Apoptosis↓	(200)
	MIF	BMMSCs	Rat/MI	Regulated the AMPK signaling	Apoptosis↓, cardiac remodeling↓	(140)
	GATA-4	BMMSCs	Mice/MI	-	Apoptosis↓, cardiac vessel density↑ c-kit-positive cells↑	(201)
	HIF-1α	BMMSCs	Rat/MI	Increased VEGF and PDGF protein	Angiogenesis↑, fibrosis↓	(202)
	CSTSMLKAC	BMMSCs	Mice/MI	-	Apoptosis↓, inflammation↓ Angiogenesis↑, fibrosis↓	(203)
Membrane modification	CMP	BMMSCs	Mice/I/R	Decreased the levels of cTnI, CK-MB, TNF-α, and IL-1β, Bax, upregulated Bcl-2 expression	Apoptosis↓, inflammation↓	(204)
	DSPE-PEG-NHS EHBPE, CP05 peptide thiolated HA-SH	UCMSCs	Rat/MI-IR	Upregulated proteins Cx43, Ki67, CD31, and α-SMA and genes *VEGFA, VEGF-B*, *vWF*, Serca2a, downregulated genes *TGF-β1*, *MMP-9*	Fibrosis↓, angiogenesis↑ Cell-to-cell interactions↑ Cell proliferation↑	(205)
	IMTP	BMMSCs	Mice/MI	Suppressed genes *p53* and *BAK1*	Apoptosis↓	(206)
Encapsulation	miR-590-3p	BMMSCs	Rat/MI	Inhibited the expression of *Clic5, Homer1 and Hopx* gene	CMs proliferation↑	(195)
	miR-19a/19b	BMMSCs	Mice/MI	Reduced the expression of *Bim* and *PTEN* genes, decreased the collagen I and III levels	Fibrosis↓, cardiac HL-1 cells apoptosis↓	(185)
	miR-126, miR-146a	ADMSCs	Rat/AMI	Upregulated CD31 and Cx43	Angiogenesis↑, fibrosis↓	(207)
	miR-21	MSCs	Mice/I/R	Decreased cleaved caspase-3, IL-6, RANTES, and IL-1α, increased IL-13	Apoptosis↓,inflammation↓	(208)
Hybridization	Monocyte mimics	BMMSCs	Mice/MI/RI	Via Mac1/LFA1-ICAM-1 interaction	inflammation↓, M2 ϕ↑ ECs maturation↑	(209)
	SαV-NVs,PLT-NVs	MSCs	Mice/I/R	–	Inflammation↓, apoptosis↓	(210)
	Macrophage membranes	MSCs	Mice/I/R	Eliminated ROS	Angiogenesis↑, M2 ϕ↑, inflammation↓ inflammation↓, angiogenesis↑	(211)
Biomaterials	PGN hydrogel	UCMSCs	Rat/MI	Decreased the expression of TGF-β1, TNF-α	Fibrosis↓, apoptosis↓ inflammation↓, angiogenesis↑	(212)
	(RADA)4-SDKP Hydrogel	BMMSCs	Rat/MI	Decreased the expression of TGF-β1, TNF-α	Fibrosis↓, apoptosis↓ inflammation↓, angiogenesis↑	(213)
	PPY-CHI Hydrogel	EnMSCs	Rat/MI	Increased EGF/PI3K/AKT signaling	Angiogenesis↑, apoptosis↓ alleviated arrhythmia	(214)
	Angiogenin-1 hydrogel	MSCs	Mice/MI	Increased the expression of genes *FGF*, *PGF*, and *VEGFB*	Angiogenesis↑	(215)
	RGD hydrogels	UCMSCs	Rat/AMI	Upregulated miR-221–3p expression	angiogenesis↑, apoptosis↓	(216)
	Alginate hydrogel	BMMSCs	Rat/MI	Increaed the levels of HGF, VEGF, and PDGF-BB Via polarizing M2 ϕ	apoptosis↓, angiogenesis↑ inflammation↓	(217)
	GelMA, HA loaded with CAT	MSCs	Rat/AMI	Eliminated ROS and generated O2	CMs displayed mitotic activity, increased capillary density	(218)
	OHA-PL hydrogel	ADMSCs	Rat/MI	–	ROS↓, angiogenesis↑, apoptosis↓ inflammation↓, fibrosis↓	(219)
	Peptide hydrogel+	MSCs	Pig/MI	Reduced IFNγ, TNFα, and IL12p40	Angiogenesis↑, inflammation↓	(220)
	Acellular cardiac scaffolds	MSCs	Pig/MI	Increased IL-1ra, IL-10, TGF-β3, MMP2, TIMP1 IsoB4, reduced TNFα, CCL-2 ,GM-CSF	Inflammation↓, fibrosis↓, angiogenesis↑	(221)
	MN Patch	UCMSCs	Mice/MI	Decreased IL-1β, IL-6, TNF-α, iNOS, Col-I, Col-III, MMP-2, and MMP-9 levels	Inflammation↓, fibrosis↓	(222)
	Blended PCL/COL-1 nanofibrous patch+TGF-β3	UCMSCs	Rat/AMI	–	Angiogenesis↑, fibrosis↓, apoptosis↓	(223)
	Fibrinogen	MSCs	Mice/AMI	–	NRCM proliferation↑ angiomyogenesis↑, fibrosis↓	(224)

EHBPE, hyperbranched epoxy macromer; α-SMA, alpha-smooth muscle actin; vWF, von Willebrand factor; CCL2, chemokine (C-C motif) ligand 2; IRAK1, IL-1Rassociated kinase 1; CBSC, cortical bone-derived mesenchymal stem cell; SOD, superoxide dismutase.

### 5.1 Preconditioning

The production and therapeutic properties of EVs are markedly influenced by the tissue source, donor cells and culture conditions. Preconditioning can help engineering specific MSC-EVs. Preprocessing can be achieved by exposing MSCs to drugs, cytokines, physiological stresses. Specific treatments include atorvastatin, hemin, tongxinluo, tanshinone IIA, LPS, C1q-TNFα related protein-9 (CTRP9), hypoxia, and three-dimensional (3D) cell cultivation (188–194, 206, 225). Preconditioning typically moduate the secretome of MSCs with altered cytokines, chemokines, enzymes, or growth factors secretion, as well as influence the EVs synthesis process to enrich specific miRNA in MSC-EVs. Platelet-derived growth factor (PDGF)-BB is a potent mitogen of MSCs, enhancing the cardioprotection of MSCs by suppressing the expression of miR-320 (226). Moreover, miRNAs regulated by preconditioning also affect the survival of MSCs and function of MSC-EVs. Ischemic preconditioning can induce the expression of miR-107 in MSCs, thereby significantly improving transplanted MSCs in infarcted myocardium (227). Compared with normoxia-conditioned BMMSC-EVs, hypoxia-conditioned BMMSC-EVs exhibited elevated expression of miR-125b-5p (206) and miR-210 (188), which reportedly facilitate ischemic cardiac repair by reducing CMs apoptosis. Low oxygen levels triggered the production of HMGB1 in BMMSC-EVs, which promotes angiogenesis via JNK/HIF-1α signaling (228). Studies have shown that IFNγ and hypoxic pretreatment can induce partial changes in miRNA in EVs in a donor dependent manner, but their effects are far less important than their impact on protein content (229). Preprocessing is believed to overcome inter-donor variability in MSCs function. However, not all donors have similar responses to pretreatment initiation, indicating the need to test and optimize pretreatment for each individual indication, and careful selection of donors may be necessary in allogeneic therapy. In addition, attention should be paid to the degree of hypoxia. Moderate hypoxia (3%–5% O2) has been shown to stimulate MSCs proliferation (230). However, a sharp decrease in oxygen tension (<1%) potentiated a glycolytic metabolism and cell quiescence (230).

By simulating the physiological environment of tissue morphology and intercellular interactions *in vivo*, 3D cultures can influence the biogenesis and function of EVs (231). The two primary categories of 3D cultures are static (e.g., hydrogels and fiber scaffolds) and dynamic (e.g., perfusion bioreactors and microcarrier-based stirred bioreactors) (232). Cultivating UCMSCs in scalable microcarrier-based 3D cultures has been found to result in an approximately 20-fold increase in EVs production when compared with two-dimensional (2D) cultures. Moreover, the combination of tangential flow filtration and 3D cultures can further enhance the EVs yield by 7-fold, resulting in a 7-fold improvement in the transfer of small interfering RNA (siRNA) to neurons. This evidence demonstrates the synergistic enhancement in the EVs yield and transport properties (233). Furthermore, MSC-EVs obtained from 3D cultures were found to exhibit enhanced immunomodulatory potential, as evidenced by previous studies (234, 235). Furthermore, a hollow-fiber bioreactor-based 3D cultures system has been proven to considerably boost the production of MSC-EVs, resulting in robust cardioprotective effects in rats with AMI (194).

### 5.2 Gene engineering

Gene engineering can adjust the expression and release of EVs in MSCs, allowing for targeted delivery to specific tissues. Transduction of lentivirus, plasmid, and adenovirus vectors into parental cells are successful methods for selectively altering the composition of MSC-EVs (236). EVs released from BMMSCs overexpressing miR-30e can improve myocardial injury, inhibit myocardial cell apoptosis and cardiac fibrosis after MI in rats (196). In non-human primate (NHP) MI models, EVs produced by MSCs overexpressing miR-486-5p demonstrated substantial improvements in cardiac function and angiogenesis, with no increase in the incidence of arrhythmia-related complications (197). Hu etal. have demonstrated that EVs derived from MSCs overexpressing islet-1(ISL1) (ISL1-MSC-EVs) have the independent ability of EC-protective and pro-angiogenic and angiogenin-1 hydrogel can retain ISL1-MSC-EVs in ischemic heart, improving the survival and angiogenesis of ECs and promoting heart repair (215). GATA-4-expressing BMMSC-EVs can induce BMMSCs differentiation into CM-like cells, reduce hypoxia-induced CMs apoptosis, and improve myocardial function post-MI (201). Studies have shown that EVs from MSCs with overexpressing HIF-1α has been found to enhance neovascularization and suppress myocardial fibrosis in rats with MI (202). In a rat model of AMI, EVs secreted by MSCs overexpressing AKT showed higher levels of PDGF-D, which promoted post-MI angiogenesis and substantially improved cardiac function (198). Furthermore, PDGF could stimulate ADMSCs to secrete EVs carrying c-kit and stem cell factors, enhancing their angiogenic capacity (237). CXCR4, a G-protein-coupled receptor, in conjunction with stromal cell-derived factor (SDF)-1α serves as a major regulator of stem/progenitor cell activities. CXCR4-enriched MSC-EVs have been found to reduce MI-induced cell death and promote angiogenesis by activating the PI3K/AKT signaling pathway both *in vitro* and *in vivo*. This finding suggests that CXCR4 plays a pivotal role in angiogenesis (199). Moreover, overexpression of SDF1 in MSC-EVs suppressed autophagy of ischemic CMs and promoted microvascular production of ECs (200).

Genetic manipulation of parental cells represents a method to obtain engineered EVs with target characteristics by recombining functional peptides with EVs membrane proteins or lipid-binding proteins/peptides and displaying functional peptides on the EVs surface (238). Lysosome-associated membrane protein 2b (Lamp2b) is the most frequently used membrane protein for decorating EVs with targeting moieties. Wang et al. fused the ischemia-targeting peptide (IMTP) CSTSMLKAC with Lamp2b and introduced it into MSCs through lentivirus-based vector. This substantially enhanced the targeting ability of EVs to both hypoxia-injured H9C2 cells and the ischemic myocardium, thereby suppressing inflammation and CMs apoptosis, reducing infarct size, and improving cardiac function in mouse MI models (203). In terms of peptides that cannot be effectively displayed on the EVs surface upon fusion with Lamp2b, the introduction of a glycosylation sequence at a specific position in the engineered fusion protein may enhance stability (239). Based on the high levels of cardiac troponin I (cTnI) detected in the infarct area, Wang et al. expressed a cTnI-targeted short peptide on the surface of MSCs through gene transfection to obtain cTnI-targeted EVs. Furthermore, hsa-miR-590-3p was incorporated into cTnI-targeted EVs via electroporation. Upon intravenous administration, these EVs containing hsa-miR-590-3p localized to the infarct area along the cTnI concentration gradient and were endocytosed by CMs, thereby promoting CMs proliferation in the peri-infarct area and improving cardiac function (195).

### 5.3 Membrane modification

The membrane modification of EVs can be achieved through methods such as click chemistry and lipid insertion. Lipophilic components can be easily inserted into the membrane. Especially, distearoyl phosphoethanolamine (DSPE) can be embedded into the phospholipid bilayer, thereby anchoring the attached components to the EVs surface. In order to protect CM specific peptides (CMP, WLSEAGPVVTVRALRGTGSW) from degradation, Gu et al. modified CMP with covalently bound 1, 2-distearoyl-sn-glycero-3-phosphoethanolamineN-[hydroxysuccinimidyl (polyethylene glycol)-2000] (DSPE-PEG-NHS), and then linked the PEG modified protein peptide to the EVs. Subsequently, the miR-302 mimic was loaded into the engineered EVs using electroporation technology. Compared with unmodified EVs, engineered EVs can be more effectively taken up by CMs, promote CMs proliferation *in vitro*, reduce CM apoptosis and inflammatory response, and improve cardiac function after myocardial I/R injury (204). Targeting peptides or fluorescent molecules can be decorated on EVs surface through the click chemistry with these groups. Zou et al. synthesized a hyperbranched epoxy macromer grafted with an aniline tetramer to cross-link thiolated hyaluronic acid and thiolated UCMSC-EVs anchoring a CP05 peptide via an epoxy/thiol “click” reaction. The resulting Gel@Exo systemcan significantly result in a prominent therapeutic effect on MI-I/R (205). Zhu et al. conjugated EVs with a IMTP by bio-orthogonal chemistry, which showed specific targeting to the ischemic area and exerted a significant cardioprotective effect post-MI (206).

### 5.4 Encapsulation of medicinal agents

The methods for EVs loading mainly include incubation, electroporation and permeabilization (240, 241). Suitable packaging methods can be selected based on the properties of the packaging molecules. Small molecules can be introduced into EVs through incubation or ultrasound assistance. Macromolecules need to enter EVs by electroporation. Sun etal. have demonstrated that curcumin transported via EVs remains more stable and achieves higher concentrations in the bloodstream (242). Wang et al. used electroporation to load miR-590-3p into EVs for systemic administration in animal models of MI (195). However, it is important to note that this method may induce the formation of siRNA aggregates, which can affect loading efficiency (243). In a mouse model with MI, the combination of EVs loaded with miR-19a/19b and MSCs transplantation significantly promoted the repair of infarcted heart (185). In addition, ADMSC-EVs can be an effective nanoshuttle for miR-126 and miR-146a (207). CD47 enables cells to evade clearance by macrophages through CD47-signal regulatory protein α binding. EVs were isolated from MSCs overexpressing CD47 (CD47-EVs) and then loaded with miR-21a via electroporation, resulting in electro CD47-EVs. Exogenous miR-21 was efficiently internalized into CMs, leading to inhibition of apoptosis, reduced inflammation, and improved cardiac function after myocardial I/R (208).

### 5.5 Hybridization

EVs can readily be fused with other types of exogenous lipid membrane structures by extrusion, filtration or freeze-thaw cycles methods (244). This fusion combines the advantages of liposomes and EVs, improving their circulation stability and drug delivery efficiency (245, 246). Distinct from the original EVs, a kind of hybrid EVs with liposomes can be used as delivery systems for larger cargoes, such as CRISPR/Cas9 (247), expanding the scope of applications of EVs as drug delivery systems. Zhang et al. modified MSC-EVs with monocyte membrane by an incubation-extrusion method, resulting in hybrid EVs with regenerative potential of stem cells and inflammatory targeting characteristics of monocytes. The interaction between macrophage receptor 1 (Mac1)/lymphocyte function-associated antigen 1 (LFA1)-intercellular cell adhesion molecule-1 (ICAM-1)-induced adhesion and transmigration at least partly levered the targeting efficiency. This modification markedly enhanced the homing efficiency of EVs in injured hearts and effectively alleviated myocardial I/R injury in mice (209). Recently, Lai et al. (210) developed genetically modified hybrid nanovesicles (hNVs) that include cell-derived nanovesicles with high-affinity SIRPα variants (SαV-NVs), MSC-EVs, and nanovesicles from platelets (PLT-NVs). SαV-NVs suppressed CD47-SIRPα interaction, promoting macrophage phagocytosis of dead cells. EVs components can alleviate inflammation. Furthermore, PLT-NVs provide hNVs the ability to evade immune surveillance and selectively target the infarct areas. The combined effects of hNVs notably improved the LVEF on day 21 in an I/R mouse model, offering a simple, safe, and robust strategy for boosting cardiac repair. The liposome-based cellular engineering method can be achieved by engineering parental cells with membrane fusogenic liposomes to equip EVs with various functional agents, including drugs, fluorophores and bio-orthogonal chemicals (248). Zhang et al. constructed a hybrid cell-derived EVs (N@MEVs) that was composed of MSCs and macrophage membranes encompassed MitoN, a ROS scavenger, to boost the healing of the heart. Fixing l-arginine within N@MEVs further enhanced the potential for delivery to injured cardiac tissues. This combination therapy has demonstrated synergistic effects on cardiac repair and regeneration, specifically via the regulation of M2 ϕ, promotion of angiogenesis, and reduction of DNA damage, thereby restarting CMs proliferation (211).

### 5.6 Biomaterial-assisting

The therapeutic potential of MSC-EVs in cardiac repair has been constrained by limitations such as poor retention, brief biological half-life, necessity for repeated administration, and risk of secondary tissue damage (249). With the rapid development of biomaterials and tissue engineering, the combination of EVs with biomaterials can compensate for the shortcomings of EVs in specific applications of tissue repair. Injectable, biocompatible, hydrophilic, tunable, conductive, and compositionally versatile hydrogels serve as intriguing platforms for replicating cardiac ECM (250). In addition, hydrogels provide a controllable delivery system depending on the type of substrates used in their structures. Hydrogels can be used as injectable matrix for direct injection into injured myocardium and as myocardial patch placed on the surface of injured area. Polymer-based hydrogels infused with EVs, including hyaluronic acid (HA), gelatin, chitosan, silk, and alginate, are utilized to enhance cardiac healing (251). The use of hydrogels can enhance EVs stability and delivery to a specific injury site in a controlled and adjustable manner while enabling sustained *in situ* release.

Han et al. used a self-assembled peptide hydrogel (PGN hydrogel) to encapsulate UCMSC-EVs. Administering the EVs/PGN hydrogel mixture into the infarcted area improved cardiac function, as evidenced by a reduction in inflammation, fibrosis, and apoptosis, as well as an increase in angiogenesis (212). In rats with MI, cardiac function was improved after IM injection of MSC-EVs alone or in conjunction with (RADA)4-SDKP hydrogel (213). Following MI, scar formation in and around the infarction disrupts electrical signal propagation, leading to desynchronized cardiac activation and contraction (252). Conductive hydrogels have the potential to restore electrical impulse propagation during MI, preventing arrhythmias and protecting ventricular function (253, 254). Zou et al. formulated an injectable conductive hydrogel containing thiolated CP05 peptide to anchor UCMSC-EVs. The electrical activity of the hydrogel effectively improved the polarization of connexion 43 (Cx43) in cell-cell interactions, suppressing the risk of arrhythmias. In addition, the hydrogel enhanced EVs retention, consequently improving cardiac function and promoting vascular regeneration after I/R (205). Yan et al. developed an injectable hydrogel by incorporating EnMSC-EVs into polypyrrole chitosan (PPY-CHI). This synergistic combination of EVs and PPY-CHI improved cardiac function, as evidenced by the promotion of angiogenesis, inhibition of cell apoptosis, and resynchronization of electrical conduction (214). EVs from MSCs overexpressing HIF-1α can promote the angiogenesis and the apoptosis of CMs via upregulating the expression of miR-221-3p. RGD hydrogels can enhance the therapeutic efficacy of HIF-1α engineered MSC-EVs (216). IM injection the sodium alginate hydrogel incorporated with MSC-EVs enhanced the reparative potency of MSC-EVs in pro-angiogenesis, reducing fibrosis and improving cardiac function after MI (217). Recently, Wang et al. have discovered that MSC-EVs encapsulated gelatin methacryloyl/HA blended and oxygen releasing injectable hydrogel by CMs induction and vascularization in rat MI model (218). Ren etal. have demonstrated that an injectable ADMSC-EVs loaded HA-polylysine hydrogel for cardiac repair via modulating oxidative stress and the inflammatory microenvironment after MI (219).

Decellularized cardiac scaffold is a source of biological ECM derived from natural heart tissue, with conserved ECM structures and functional cardiac ECM components (255). The combination of MSC-EVs and decellularized heart tissue represents a hopeful tissue engineering approach, capable of locally administering MSC-EVs and boosting their therapeutic impact to recover cardiac function post-MI. Porcine heart adipose tissue-derived MSC-EVs (cATMSC-EVs) and peptide hydrogels were embedded in acellular porcine pericardial scaffolds for local myocardial delivery. Subsequently, the engineered scaffolds were administered to the ischemic myocardium of a pig model of MI. Six days after implantation, the designed scaffolds integrated into the infarcted heart tissue, resulting in an increase in vascular density and a reduction in macrophage and T-cell infiltration within the damaged myocardium (220). The same research group subsequently evaluated the long-term functional impact of cATMSC-EVs acellular cardiac scaffolds in a porcine MI model. The authors discovered that cATMSC-EVs enhanced post-MI right ventricular ejection fraction and ventricular dilation while also alleviating adverse cardiac remodeling. Remarkably, cATMSC-EVs also modulated the expression of inflammatory mediators and fibrosis modulators (221).

Cardiac patches provide an efficient method for administering treatments directly to the cardiac tissue. Yuan et al. designed a biocompatible gelatin-based microneedle (MN) patch loaded with UCMSC-EVs containing miRNA-29b mimics. The implantation of the MN patch into the infarcted hearts of mice resulted in increased EVs retention in the infarcted area, reducing inflammation, infarct size and fibrosis, and improving cardiac function (222). Guan et al. developed a blended polycaprolactone/type I collagen (PCL/COL-1) nanofibrous patch loaded with TGF-β3 and UCMSC-EVs (Exo@TGF-β3@NFs). Exo@TGF-β3/NFs upregulated genes involved in angiogenesis and mesenchymal differentiation *in vitro*. Four weeks post-transplantation, Exo@TGF-β3@NFs resulted in elevated LVEF and fraction shortening *in vivo*. Furthermore, Exo@TGF-β3@NFs could substantially reduce the size of MI, inhibit fibrosis, and increase scar thickness (223). Yao et al. designed and tested a minimally invasive EVs spray using MSC-EVs and biomaterials. In a mouse model of AMI, administration of this spray via thoracoscopy improved cardiac function, reduced fibrosis, and promoted endogenous angiomyogenesis in the post-MI heart. This delivery method was found to increase the retention of EVs and reduce surgical stress and inflammatory responses (224). Born et al. have demonstrated that MSC-EVs can be incorporated into a 3D-printed gelatin methacrylate (GelMA) hydrogel bioink while retaining their bioactivity. By increasing the crosslinker concentration, the initial burst release of EVs can be reduced during gelation (256).

## 6 Clinical trials of EVs in MI or I/R

Due to the inherent complexity of its mechanism of action, the application of EVs in the treatment of CVD has considerable appeal. EVs are being examined in clinical trials to assess their safety and efficacy as therapeutic agents. Nevertheless, clinical trials of EVs for cardiac indications are still in their early stages, and evidence supporting their clinical application in patients with MI or I/R is limited. As of August 2024, based on information from https://clinicaltrials.gov/ (Table 2). An ongoing trial (NCT05669144) is investigating the combined transplantation of mitochondria and administration of MSC-EVs in candidates for coronary artery bypass grafting (CABG) surgery. Patients in the experimental group will receive co-transplantation IM and IC injection of EVs (1 ml of EVs containing 100 μg of EVs) and mitochondria(1 ml of EVs containing 10 million mitochondria). Twenty patients will be recruited and the evaluation of the patient's recovery will be performed 1 month after the surgery. There are also some studies about EVs and MI or IR. A single-center study in the United States (NCT04327635) is aiming to assess the safety of purified EVs (PEP) derived from stored human blood in patients undergoing coronary stent implantation. Twelve patients who undergo PCI will be treated with a single dose of PEP within 20 min after stent placement or post-dilation. The study will conduct a one-year follow-up to evaluate the dose-limiting toxicity and maximum tolerated dose of PEP with increasing concentrations of EVs. In addition, the study plans to assess infarct size and EF, as well as monitor the alloimmune response. A French study (NCT05774509) plans to evaluate the safety and efficacy of three IVs of EVs-enriched secretome (20 × 10E9 particles/kg for each infusion) of CPCs in severely symptomatic patients with drug-refractory left ventricular dysfunction secondary to non-ischemic dilated cardiomyopathy. Another registered trial (NCT04127591) aims to determine the expression profile of miRNAs in peripheral blood EVs of patients with MI and investigate their relationship with MI. Furthermore, a multicenter observational prospective study (NCT06070974) plans to assess the potential of plasma EVs in identifying patients at a high risk of adverse remodeling following STEMI at an early stage. This could facilitate appropriate patient management and reduce the risk of cardiovascular events. Consecutive patients with STEMI will be enrolled three days after PCI to investigate the correlation between the EVs profile and the severity of MI. A Phase IV trial (NCT02931045) examined the concentrations of platelet EVs, c-reactive protein, IL-6, and elastase in patients after 6 months of antiplatelet therapy with ticagrelor or clopidogrel. The objective of this study is to identify an additional mechanism of the action of ticagrelor, which might contribute to the observed clinical benefits in patients treated with ticagrelor. Although the safety of cardiac or intravenous injections of EVs has been assessed in clinical trials, notable concerns regarding the safety and tolerability of repeated injections remain.

**Table 2:** Clinical trials of extracellular vesicles for cardiac indications (clinicalTrials.gov).

Conditions	Interventions/treatment	Status	Study type	Sponsor	Clinical trial number
Myocardial infarction Myocardial ischemia Myocardial stunning	Biological: Mitochondria and MSC-derived exosomes	Recruiting	Interventional	Tehran University of Medical Sciences	NCT05669144
Percutaneous Coronary intervention	Drug: PEP	Enrolling by invitation	Interventional	Christopher J. McLeod	NCT04327635
Myocardial infarction	Other: exosome	Unknown status	Observational	Xinhua Hospital, Shanghai Jiao Tong University School of Medicine	NCT04127591
STEMI	Diagnostic Test: CMR and blood collection	R ecruiting	Observational	Centro Cardiologico Monzino	NCT06070974
Myocardial Infarction	Drug: Ticagrelor Drug: Clopidogrel	Completed WITH RESULTS	Interventional	Medical University of Warsaw	NCT02931045
Heart failure with reduced ejection fraction	Biological: extracellular vesicle-enriched secretome of cardiovascular progenitor cells differentiated from induced pluripotent stem cells	Recruiting	Interventional	Assistance Publique- Hôpitaux de Paris	NCT05774509

CMR, cardiovascular magnetic resonance.

Compared with parental cells, MSC-EVs have several potential advantages: relative safety, because they cannot replicate and their smaller size allows them to pass through capillaries without clogging (257); low immunogenicity and easy to store, they can be stored at −20°C for up to 6 months without significant damage (258); they can transit physiological barriers, such as the blood-brain barrier (259). However, there are still several factors to be addressed for effective clinical translation, including production, isolation, dosage and administration method of MSC-EVs (260). In addition, although the safety of cardiac or intravenous injections of EVs has been assessed in clinical trials, notable concerns regarding the safety and tolerability of repeated injections remain. Improving the biological benefits of MSC-EVs by engineering parental cells or post-production EVs and improving the delivery of EVs by encapsulation in biomaterial to prolong their efficacy, which may help to promote their clinical translation.

## 7 Conclusions

Over the past decade, significant progress has been made in the understanding of the biology of EVs and their important role in cardiovascular pathology and physiology. As multifunctional carriers of molecular signals, EVs from cardiac cells can either transmit both protective and damaged signals in MI. Moreover, it has been well-recognised that MSC-EVs have great potential in the treatment of MI, potential mechanisms including promoting angiogenesis, mitigating inflammation and oxidative stress, inhibiting cell death and improving cardiac remodeling, typically indicating that EVs are as effective as their parental cells.

However, there are still many challenges such as the isolation and characterization of clinical grade biological products EVs, scalable production, and batch standardization. Maldistribution of EVs within the body after systemic administration is also a challenge for achieving targeted drug delivery. In addition, despite preclinical studies on EVs demonstrating the apparent lack of immunotoxicity, immunological clearance remains largely unexplored. The rapidly growing EVs therapeutics and drug delivery systems requires an understanding of undesired immunogenicity, which is critical for the development of safe and efficient clinical products. The use of immortalized cell lines can minimize variability of EVs to improve the reproducibility of clinical outcomes. We know little about how cardiovascular pathophysiology changes the EVs biology, how EVs derived from different types of myocardial cells mediate intercellular communication in damaged myocardium and how MSC-EVs are designed to maximize their beneficial effects in the hypoxic/ischemic microenvironment. It is still unclear whether specific promotion or inhibition of EVs production will be beneficial for MI. The progress in these fields not only helps to reveal previously unknown mechanisms of CVDs, but also lead to new treatment methods to improve clinical outcomes. The development of technologies could provide novel insights into EVs biology and the clinical applications of MSC-EVs, paving the way for developing personalized precision medicine.
